# Supplementary material for: Are carbon emissions trading and green financial instruments synergistic? -Comprehensive quantitative research based on content analysis
Source: PLoS One. 2024 Mar 7;19(3):e0298601. doi: 10.1371/journal.pone.0298601 (PMC10919601; doi:10.1371/journal.pone.0298601)
Supplement: S1 Data — (ZIP) [file pone.0298601.s001.zip › Data/地级市Z_F工作报告.zip!/║╙─╧/╞╜╢Ñ╔╜╩╨/└┤╫╘╣1⁄2╓┌║┼í╛┬φ┐╦╩2╛▌═°í┐_2011.docx]

各位代表：

现在，我代表市人民政府，向大会报告政府工作，请予审议，并请各位政协委员提出意见。

一、2010年及“十一五”工作回顾

2010年，面对复杂多变的经济环境和困难局面，市人民政府在省委、省政府和市委的正确领导下，团结带领全市人民，深入贯彻落实科学发展观，加快转变经济发展方式，学先进、比创新、看实效，较好地完成了市九届人大二次会议确定的各项目标任务。

初步预计，全市生产总值达到1300亿元，比上年增长12.1%；地方财政一般预算收入80.6亿元，增长14.6%；规模以上工业增加值669.6亿元，增长15%；全社会固定资产投资710亿元，增长22.9%；社会消费品零售总额350亿元，增长19.5%；城镇居民人均可支配收入16208元，增长10.1%；农民人均纯收入5504元，增长15.2%。

一年来，我们主要抓了以下重点工作:

(一)坚持多策并举，保持了经济平稳较快发展。抓住需求不足这一突出矛盾，把扩大投资、促进消费、服务企业作为关键性举措，加强经济运行调节，取得了扩内需、保增长的明显成效。重点项目建设加快推进。170个重点项目完成投资178.5亿元，占全年预期投资的119%。中平能化碳化硅微粉、500千伏平南输变电等48个项目建成投产(投用)，特别是鲁阳电厂一期2×100万千瓦机组的并网发电和叶舞高速的建成通车，使我市的发电装机容量达到560万千瓦，高速公路通车里程达到396公里，实现县县通高速，为全省发电装机容量突破5000万千瓦、高速公路通车里程突破5000公里作出了积极贡献，具有里程碑的意义。平煤机高端液压支架、中平能化两万吨高性能浸胶帘子布、东方希望集团盐化工等50个项目开工建设，新平高工程、泓利焦化年产90万吨捣固焦及综合利用等37个续建项目加快推进，为今后发展蓄积了后劲。消费需求较快增长。加快“万村千乡市场工程”和“新网工程”建设，新增农家店305家，新建农资配送中心6个、连锁店190个。全面落实扩大消费政策，家电下乡和汽车摩托车下乡产品销售量、补贴额一直保持全省领先位次，带动社会消费品零售总额增速位居全省前列。企业服务长效机制初步建立。市、县两级各抓100家重点企业，健全政策导向、要素保障、困难企业帮扶等十项企业服务长效机制。强化财税收支管理，落实税收优惠政策，减轻了企业负担。积极推动银企合作，全年融资总额178亿元，其中新增贷款135亿元。加强煤电油气运协调，有效保障了生产要素供给。

(二)坚持政策引导，推进了产业结构优化升级。出台了加快战略性新兴产业和产业集聚区发展的政策措施，引导产业结构优化升级。支柱产业优势继续巩固。实施工业结构转型升级项目72个，完成投资84.7亿元，能源化工、装备制造、特种钢材、新型建材、农产品加工等支柱产业规模扩大、技术提升，对经济增长的贡献率进一步提高。中平能化集团营业收入超过1000亿元，巩固了平顶山能源、盐化、焦化、尼龙化工等主导产业在全国乃至世界的重要地位。现代服务业加快发展。在全省率先制定了旅游目的地建设规划，实施了一批文化旅游基础设施项目。加大宣传推介和景区创建力度，开展了全国首例景区错峰免门票活动，全年接待游客、旅游总收入均增长35%以上。完善金融服务体系，广发银行、兴业银行、交通银行入驻我市；市商业银行升级更名为平顶山银行，实现了由地方银行向区域性银行的转型。产业集聚区建设明显提速。10个省级产业集聚区完成基础设施投资42亿元，增长122.7%；完成工业投资108亿元，增长57.6%；新开工项目156个，在建亿元以上项目38个，新入驻企业96家。自主创新能力不断增强。新增省级工程技术研究中心5家、企业技术中心4家、质检中心2家、重点实验室1个。舞钢公司荣获“全国质量奖”，“神马弹力丝”、“天瑞水泥”荣膺“中国驰名商标”，行政服务综合楼工程获国家“鲁班奖”，新增省名牌产品16种、省著名商标10件，新认定国家级高新技术企业4家，高新技术产业增加值增长21.1%。节能减排和生态建设得到加强。深入开展洗煤、焦化行业综合整治，188家60万吨以下小洗煤厂全部关闭，淘汰小焦化产能120万吨。大力发展新型墙材，产能达到45亿标块。扎实推进林业生态建设，造林18.4万亩，汝州市、宝丰县、湛河区初步通过省级林业生态县验收。万元生产总值能耗下降2.92%，化学需氧量、二氧化硫排放量分别低于省定目标1.8个和4.6个百分点。

(三)坚持固本强基，巩固了“三农”基础地位。强农惠农政策全面落实。全年财政用于“三农”的投入达到23.9亿元，增长22.6%，其中发放粮食直补等八项补贴4亿元。农业综合生产能力稳步提高。32座病险水库除险加固工程和北汝河治理一期工程完工，发展有效灌溉面积6.3万亩，改造中低产田5万亩，治理水土流失面积82平方公里，新增农机总动力8万千瓦，全年粮食总产197.2万吨，连续7年获得丰收。大力发展现代畜牧业，新建标准化养殖小区286个，引进千万元以上畜产品加工项目15个。加快农业产业化步伐，新增省级龙头企业8家、农民专业合作社241个，新增土地流转面积11.6万亩。新农村建设扎实推进。新建农村公路464公里，新增沼气用户2万户，新解决12.9万人的饮水安全问题，完成了108个贫困村的整村推进和6.1万贫困人口的脱贫。培训农民工6.2万人，新增转移就业6.8万人。36个中心镇、539个中心村经济发展规划和新村建设规划修编工作全面完成，“百企帮百村”活动深入开展，112个中心村启动建设，村容村貌焕然一新，生产生活条件明显改善。南水北调丹江口库区移民安置工作多次受到国家和省表彰。县域经济实力进一步增强。

(四)坚持以创促建，提升了城镇化水平。成功创建国家园林城市，持续创建国家卫生城市，启动创建国家森林城市，全市城镇化率43.4%，提高1.6个百分点。城市扩容规划加快编制。全市土地利用总体规划获批，城市总体规划通过国家部际联席会议审查，平顶山城市新区规划、平宝叶鲁空间发展战略规划编制完成。6县(市)和石龙区的城市总体规划已完成报批。基础设施建设不断加强。老城区实施了道路改扩建、垃圾处理、天然气置换、河道治理等一批基础设施建设工程，新建改建道路13条、桥梁4座，完成投资8.7亿元。强力推进城中村和旧城开发改造，开工300.5万平方米，主体完工177.7万平方米。新城区完成城建投资21.2亿元，新增道路9.7公里、房屋建筑面积36万平方米。加强园林绿化建设，新建改建绿地49.8万平方米。积极推进和谐社区建设，市民文明素质和城市管理水平进一步提升。县城和小城镇建设稳步推进。新建市政道路15条，39座建制镇垃圾中转站投入运营，城乡面貌有新的改观。

(五)坚持改革开放，增强了经济发展动力和活力。各项改革深入推进。继续深化国有企业改革，改制面达96.7%。大力推进行政审批制度改革，市、县两级审批大厅实现同网络办公，整体提速20%以上。政府机构改革顺利完成，事业单位全部推行聘任制。文化综合执法体制和警务机制改革取得突破。全市政府举办的基层医疗卫生机构全部实施国家基本药物制度。招商选资成果丰硕。成功举办第三届华侨华人中原经济合作论坛，主动到沿海发达地区组织开展一系列招商活动。全年签约项目132个，开工52个；实际到位市外资金430亿元，增长25%；实际利用外资1.64亿美元，增长16.7%。非公有制经济进一步壮大。放宽领域，优化环境，支持引导非公有制经济加快发展。非公有制经济比重达53.8%，提高0.8个百分点。

(六)坚持改善民生，促进了社会和谐稳定。全年投入民生工程资金29亿元，比上年增加2.5亿元，“十大民生工程”如期完成。就业再就业工作得到加强。城镇新增就业12.4万人，其中失业人员再就业5万人，零就业家庭保持动态为零。社会保障体系不断完善。将8.9万城市居民和17.1万农村居民纳入城乡低保，人均月补差高于省定标准。在全省率先建立了临时救助制度。进一步完善了城镇居民医保制度，提高了新农合参保标准。宝丰县、郏县被确定为国家第二批新农保试点。加强价格管理和调控，对低收入群体进行补贴，保障了市场物价基本稳定和群众基本生活。保障性住房建设力度加大。开工建设廉租房14.6万平方米、经济适用房79.1万平方米、棚户区改造40.7万平方米、公共租赁房2.8万平方米，为4510户农村贫困家庭实施了危房改造。社会事业全面进步。制定了中长期教育发展规划和中小学幼儿园发展规划，着手解决城区中小学大班额问题，市区开工建设小学3所；强力推进职教攻坚，新建专业实训基地8个，舞钢市、郏县创建为职教强县。实施文化惠民工程，建成15个乡(镇)综合文化站和300个农家书屋，开展了“欢乐中原”和“舞台艺术送农民”等群众性文化活动。健全医疗服务体系，市疾控中心综合实验楼主体完工，开工建设新区人民医院和3所县医院、7所乡(镇)中心卫生院。创建全国白内障无障碍市和无障碍建设城市工作通过国家验收。人口计生工作连续17年全省先进，是河南唯一的国家人口计生综合改革、婚育新风进万家活动“双示范”市。社会大局保持稳定。强化以煤矿为重点的安全监管，出台了落实安全生产行政首长负责制的意见，127座地方煤矿兼并重组工作有序推进，30座小煤矿关闭到位。积极开展食品药品安全专项整治，再次被评为全省食品安全优秀城市。健全应急管理机制，完善了各类应急预案。强化社会治安综合治理，加强基层基础工作，组建了专职巡防队伍，严厉打击各类违法犯罪，刑事案件发案数下降8.8%。认真解决人民群众反映的热点难点问题，信访秩序明显好转。

(七)坚持强化保障，加强了精神文明和民主法制建设。按照“三具两基一抓手”工作要求，深入推进“两转两提”，积极开展“创先争优”活动，团结拼搏、干事创业的氛围更加浓厚。坚持依法行政，自觉接受人大及其常委会的监督，支持政协参政议政，认真办理人大代表建议和政协提案。“五五”普法顺利完成，法律援助、法律服务工作明显加强。扎实推进惩治和预防腐败体系建设，加大审计监督力度，治理商业贿赂，反腐倡廉取得新成效。广泛开展群众性精神文明创建活动，新增省级文明单位43家、市级文明村镇65个，城乡文明程度明显提升。大力开展国防教育，扎实做好双拥工作，驻平解放军和武警部队在支持地方抢险救灾、重点工程建设等方面作出重要贡献。民族、宗教、外事、侨务、对台、统计、史志、气象、地震、人防、通信、烟草、档案、盐业、新闻出版、国家安全等各项事业都取得了新成绩。

各位代表!

2010年主要任务的圆满完成，标志着“十一五”规划目标胜利实现。

过去的五年，综合实力跨上新台阶。生产总值是“十五”末的1.86倍，占全省比重提升0.51个百分点；人均生产总值达到26000元，是全省平均水平的1.12倍；地方财政一般预算收入是“十五”末的2.77倍，占全省比重提升0.43个百分点；固定资产投资五年累计达到2345亿元，是“十五”期间的4.7倍。

过去的五年，结构调整迈出新步伐。二、三产业占生产总值比重比“十五”末提升3.4个百分点，高新技术产业增加值占工业增加值比重提升16.6个百分点，非公有制经济占生产总值比重提升3.9个百分点，节能减排目标圆满完成。

过去的五年，城乡面貌发生新变化。建成区面积由不足60平方公里拓展到75平方公里，城镇化率比“十五”末提高8.4个百分点；新农村建设“两个规划”全面启动；新建改建农村公路4858公里、国省干线公路213公里、高速公路235.7公里。

过去的五年，改革开放实现新突破。大中型企业实现股权多元化，行政审批效能进入全省先进行列，社会事业领域的改革取得新进展。五年实际利用外资6.8亿美元、市外资金1295亿元，分别比“十五”期间增长1.2倍和8.1倍；连续三年成功举办华侨华人中原经济合作论坛，打造了我市招商选资重要品牌。

过去的五年，文化建设呈现新气象。建设了平顶山博物馆、文化艺术中心等标志性文化设施，举办了曲艺节、魔术节、戏剧梅花奖、书法兰亭奖大赛、“三苏杯”全国诗歌创作大赛、“尧山杯”全国摄影大赛、《歧路灯》海峡两岸学术研讨会等一系列有影响的文化活动，文化氛围日益浓厚。

过去的五年，人民生活有了新改善。城乡居民收入分别是“十五”末的1.85倍和1.95倍，城镇和农村居民恩格尔系数分别下降7个和6个百分点。在全省率先建立了全民医保、农村低保和高中阶段贫困家庭学生救助等制度。

盘点“十一五”的发展，我市生产总值突破1000亿元、金融机构存款余额突破1000亿元、电网变电总容量突破1000万千伏安、中平能化集团营业收入突破1000亿元、规模以上非公有制企业突破1000家，经济社会发展站上了新的更高平台。我们从能源原材料为主的工业结构，初步构建起以产业集聚区为载体的现代产业体系，正朝着全国重要的新型能源化工基地、现代装备制造基地挺进；我们在不断完善老城区功能的同时，又新建起一座湖滨新城，正朝着平宝叶鲁一体化发展的现代化城市挺进；我们从过去人们心目中的“煤城”，创建为中国优秀旅游城市、国家园林城市，正朝着海内外知名的旅游目的地挺进。可以说，平顶山已经进入蓄势崛起的新阶段。五年来取得的辉煌成就，书写了我市经济社会发展的崭新一页，为全面建设小康社会奠定了坚实基础，值得全市500万人民骄傲和自豪，也激励着我们去奋力谱写科学发展的新篇章!

各位代表!

这些成绩的取得来之不易，这是省委、省政府和市委正确领导的结果，是市人大、市政协积极支持的结果，凝聚着全市广大干部群众和社会各界的心血和汗水。在此，我代表市人民政府，向工作在全市各条战线的广大工人、农民、知识分子、干部、驻平部队指战员、武警官兵和公安民警致以崇高的敬意!向关心、支持我市现代化建设的各界人士和国际友人、港澳台同胞、海外侨胞表示衷心的感谢!

回顾去年及“十一五”的工作，我们深刻体会到，加快平顶山的发展，必须时刻牢记发展第一要务，解放思想，抢抓机遇，奏响科学发展、加快转变经济发展方式的主旋律；必须科学谋划，用鼓舞人心、符合实际的发展思路和目标，统一思想，凝聚力量，形成率先实现崛起、提前全面小康的强大合力；必须坚定不移地推进改革开放，创新体制机制，以改革破难题，以开放带全局，增强经济社会发展的动力和活力；必须正确处理改革发展与安全稳定的关系，在安全稳定中推进改革发展，通过改革发展促进安全稳定；必须转变干部作风，狠抓工作落实，上下一心、合力攻坚，卓有成效地把各项工作推向前进；必须着力民生民心，持续加大民生投入，尽心尽力解决好关系人民群众切身利益的问题，让人民群众得到更多的实惠。

在肯定成绩的同时，我们也清醒地看到，政府工作还有不少缺点，经济社会发展中还存在一些不容忽视的矛盾和问题：一是保持经济持续较快发展难度加大。国际金融危机以来，我市部分经济指标增速下滑，在全省位次有所后移；钢铁、有色、化工等行业市场需求不足，生产经营形势比较困难；粮食稳定增产和农民持续增收基础不牢；项目谋划不足，投资总量偏低。二是经济结构性矛盾突出。高耗能、高排放行业比重较大，高技术产业和第三产业比重偏低，节能减排和结构调整任务艰巨。三是安全生产形势依然严峻。一些地方和干部安全生产责任落实不到位，发生了重特大煤矿安全事故，给人民群众生命财产造成重大损失，教训极为深刻。随着地方煤炭企业兼并重组完成后相继复工复产，安全监管压力加大。四是民生改善任务繁重。城乡公益事业和基础设施欠账较多，群众对就业、就学、就医、社会保障、住房保障等方面还不尽满意，部分低收入群众生活比较困难。五是行政效能有待进一步提高。政府职能和作风转变还不到位，个别部门和工作人员依法行政观念和为民服务意识淡薄，办事拖拉，形式主义、官僚主义仍然不同程度地存在。对此，我们一定高度重视并认真加以解决，绝不辜负人民群众的期望。

二、“十二五”发展的总体要求和奋斗目标

“十二五”时期是我市在全省率先实现崛起、提前全面小康的决胜时期，也是深化改革开放、加快转变经济发展方式的攻坚时期。根据市委七届十一次全会通过的“十二五”规划建议，市政府编制了《平顶山市国民经济和社会发展第十二个五年规划纲要(草案)》(以下简称《纲要(草案)》)，提出了今后五年经济社会发展的总体要求和奋斗目标。

总体要求是：高举中国特色社会主义伟大旗帜，以邓小平理论和“三个代表”重要思想为指导，深入贯彻落实科学发展观，以科学发展为主题，以加快转变经济发展方式为主线，以富民强市为中心任务，坚持“四个重在”实践要领，学先进、比创新、看实效，继续实施“生态建市、产业立市、文化强市、和谐兴市”战略，统筹推进优化产业结构、加快城镇化、建设新农村“三大任务”，持续提升改革开放、和谐稳定、党的建设“三项保障”，在全省率先实现崛起、提前全面小康，成为中原经济区重要的战略支点。

奋斗目标是：到2015年，力争经济总量实现“一个历史跨越”，生产总值跨越2000亿元大关，达到2300亿元(2010年价)，年均增长12%左右，人均生产总值力争超过全国平均水平；地方财政一般预算收入增速略高于生产总值增速。转型升级实现“一个重大突破”，经济增长的内生机制基本确立，发展的协调性进一步增强，现代产业体系、现代城镇体系和自主创新体系基本形成，经济转型实现重大突破。结构调整实现“三个快速提升”，即实现城镇化水平的快速提升，城镇化率超过50%；实现现代服务业发展水平的快速提升，第三产业增加值比重力争达到50%左右；实现农村生产生活水平和生态环境质量的快速提升，50%的中心村基础设施基本完善、形成建设规模。经过“十二五”的努力，将平顶山建设成为资源型城市可持续发展示范区，成为全国重要的新型能源化工基地、现代装备制造基地和海内外知名的旅游目的地，成为中原经济区重要的战略支点，在全省率先实现崛起、提前全面小康。

为实现上述目标，《纲要（草案）》还提出了优化产业结构、加快城镇化、建设新农村、加快生态建市、深化改革开放、加强社会建设等6个方面的任务。总体要求、奋斗目标和主要任务是在正确把握国际国内形势，认真研究国家宏观经济政策，全面分析我市各种有利条件，深入查找存在问题与差距的基础上提出的。这些目标任务对于加快我市经济社会发展不仅是必须的，而且通过努力也是完全可以实现的。我们坚信，只要全市上下万众一心，乘势而上，就一定能够把平顶山建设得更美好，让人民生活得更幸福，就一定能够铸就“十二五”发展的新辉煌!

《纲要(草案)》及说明已提交各位代表，请予审议。

三、2011年政府工作的主要任务

今年是“十二五”发展的开局之年。总体上看，做好今年的工作，既有有利条件，也有不利因素，应该说机遇与挑战并存，机遇大于挑战。从有利条件看，世界经济将继续缓慢复苏，我国经济发展长期向好的趋势没有改变，国际国内产业转移趋势更加明显；经过“十一五”的发展，我市资源、区位和产业优势正在扩大，中平能化集团、平高集团等一批大型企业规模效益倍增，文化旅游产业发展势头强劲、潜力巨大，经济发展具有总量扩张和产业升级的强大内生动力；加之国家加大促进中部地区崛起力度和我省建设中原经济区，有利于我们发挥优势，积极承接产业转移和争取上级支持。从不利因素看，国际金融危机尚未结束，不稳定、不确定因素仍然较多；国家将货币政策从“适度宽松”转向“稳健”，强调把好流动性总闸门、有效控制信贷投放，在土地供给、项目审批、节能减排等方面也提出了新的更高的要求，经济发展的宏观环境和要素供给形势趋紧；我们面临着既要保持经济平稳较快增长，又要调结构、促转型，还要防通胀的复杂局面。必须保持头脑清醒、增强忧患意识，坚定信心、积极应对，确保“十二五”开好局、起好步。

经济社会发展的主要预期目标是：生产总值增速高于全省平均水平1到2个百分点，达到11%以上；地方财政一般预算收入增速高于生产总值增速1个百分点，达到12%以上；全社会固定资产投资增长21%；社会消费品零售总额增长16%；外贸出口增长21%，实际利用外商直接投资增长23%；居民消费价格涨幅控制在4%左右；人口自然增长率控制在6.5‰以内；城镇新增就业7万人以上；城镇化率提高1.5个百分点；第三产业比重提高2个百分点以上；城乡居民收入较快增长；节能减排完成省定目标。

围绕上述目标，重点抓好9个方面的工作:

(一)着力扩大需求，保持经济更好较快发展。坚持统筹兼顾，扩大需求规模，优化需求结构，推动经济发展由回升向好向稳定增长转变。

努力扩大投资需求。今年计划安排第一批重点项目130个，总投资1265亿元，年度完成投资130亿元。其中，工业项目82个，年度投资81.9亿元。开工建设中平能化年产40万千瓦太阳能电池用硅片、圣光医药用品制造产业园等39个项目；续建平煤机高端液压支架、恒基伟业太阳能电池等16个项目；建成平高东芝输变电设备制造、巨龙淀粉公司玉米葡萄糖生产线等21个项目；加快推进鲁阳电厂二期2×100万千瓦机组、中平能化年产20万吨己内酰胺等6个重大项目前期工作。交通项目13个，年度投资10.7亿元。开工建设焦桐高速汝州至登封段、郏县地方铁路、宝丰县至石龙区快速通道等5个项目；续建孟平铁路复线电气化改造项目；积极推进郑渝铁路客运专线平顶山段(站)、平顶山至许昌城际轨道交通、尧山旅游支线机场、沙河平顶山段复航工程及平郏、平叶、平鲁快速通道等7个项目前期工作。农林水利项目7个，年度投资16.2亿元。开工建设农村饮水安全工程、北汝河综合治理主体工程；续建南水北调工程平顶山段、昭平台灌区配套等5个项目。服务业项目5个，年度投资6.3亿元。开工建设大香山现代物流配送中心、舞钢金马钢铁加工物流配送中心；续建宝丰文化创意产业园；积极推进通用航空产业园、卫东区顺德集团仓储物流项目前期工作。城市基础设施和社会事业项目23个，年度投资14.9亿元。开工建设市第二污水处理厂、平顶山学院扩建等3个项目，续建采煤沉陷区综合治理、平西火车站广场改造、新区人民医院等12个项目；建成大香山路、蓝欣家园经济适用房等4个项目；加快南水北调受水区供水工程等4个项目前期工作。继续坚持市级领导联系重点项目、联审联批、观摩点评等制度，认真落实建设条件和配套资金，实行网络规划、节点控制，力争早开工、早投产、早见效。加强项目库建设，搞好项目筛选、论证和储备。

积极扩大消费需求。落实调整收入分配关系的各项政策，继续增加政府支出用于改善和扩大消费的比重，着力提高城乡低收入群众收入，扩大中等收入群体，增强群众消费能力。落实鼓励消费政策，培育消费热点。积极发展消费信贷，促进消费增长。大力整顿和规范市场秩序，健全市场体系和社会信用体系，维护消费者合法权益，营造放心、舒心的消费环境。

加强经济运行调控调节。依法规范税收征管和非税收入管理，提高财政调控能力。落实各项税收优惠政策，进一步减轻企业和居民负担。组织多种形式的银企洽谈会，完善激励机制，引导各类金融机构切实增加有效信贷投放。健全企业服务长效机制，市、县两级继续分别抓好100家重点企业，推动企业服务工作常态化、制度化。加强经济运行监测，做好煤电油气运协调工作，特别要保障电煤供应。加强产运需衔接，保障重要生产资料和居民生活必需品供应，坚决打击囤积居奇、哄抬价格等不法行为，确保全市价格总水平基本稳定。

(二)着力优化结构，加快转变经济发展方式。制定资源型城市可持续发展示范区建设规划，推动产业结构转型升级，提升产业竞争力。培育壮大战略性新兴产业。突出抓好新能源、新材料和生物医药等新兴产业发展，依托中平阳光、恒基伟业、易成新材料等企业发展太阳能光伏产业，打造完整的垂直光伏产业链；依托平高集团智能电网输变电设备项目和国家级高压电气设备质检中心建设，发展高端装备制造业。改造提升传统支柱产业。围绕做大做强能源化工、装备制造、特种钢材、新型建材、农产品加工等优势产业，安排实施中平能化2万吨锦纶66帘子布、平高集团特高压超高压开关装备产业基地等一批重大转型升级项目，年度投资80亿元以上，拉长产业链条，提升产业层次。加强自主创新体系建设。积极实施自主创新体系建设发展规划，增强科技进步对经济发展的支撑作用，引导创新要素向企业集聚，加快形成一批拥有自主知识产权的核心技术、知名品牌和创新型企业。申报省级以上工程技术研究中心5家、企业技术中心4家、质检中心2家、重点实验室1个。培育国家级高新技术企业6家、省级以上创新型试点企业1家，高新技术产业增加值增长15%以上。大力实施质量兴市战略和商标战略，推进标准化体系建设，培育自主品牌，提高产品质量安全水平。加快创建品牌产业集聚区。实施产业集聚区发展赶超行动计划，全年10个省级产业集聚区完成投资300亿元，实现主营业务收入800亿元，争取有2个集聚区进入全省前30名，高新区创建为国家级高新区。通过3到5年的努力，依托高新区内中平能化集团和平高集团发展，打造亚洲最大的尼龙化工基地和全国重要的现代装备制造基地；依托平顶山化工产业集聚区和叶县产业集聚区，突出发展煤化工、盐化工，利用良好的煤电产业基础，打造全国重要的能源化工基地；依托平新产业集聚区和宝丰产业集聚区，打造高端装备制造业和太阳能光伏产业基地。通过强化产业支撑，提升经济综合实力，把我市建设成为中原经济区重要的战略支点。

(三)着力发展现代服务业，推动第三产业全面提速。继续实施现代服务业发展提速工程，加快壮大服务业规模，全面提高发展水平。大力发展优势服务业。编制实施全市文化旅游产业发展规划，加快宝丰文化改革发展实验区建设，再规划建设一批特色文化产业园区。全面启动大香山风景名胜区项目，确保香山寺保护性建设主体工程完工、大香山国学堂等项目开工。加大佛泉寺景区和温泉开发建设力度。积极开发汝瓷、金镶玉等地方特色文化旅游产品，拓展文化市场，壮大文化产业规模。大力推进景区创A工作，尧山景区创建为国家5A级景区。加大旅游基础设施建设力度，启动市旅游服务中心、白龟湖国家湿地公园等项目建设。进一步整合旅游资源，精心策划设计旅游宣传产品，提升整体形象和实力，着力打造海内外知名的旅游目的地。以孟平铁路复线建设为契机，完善沿线场站设施功能，提高物流配送能力；开工建设开源路蔬菜批发市场，加快建设市中心货运站、平西综合物流园区，建成平东煤炭物流中心等项目。适应消费升级需要，积极运用先进技术和经营方式改造提升传统商贸业，进一步会聚人气、商气，营造商机，加快打造豫西南商贸中心。着力发展高成长性服务业。抓住金融机构加速在我市布局的机遇，争取浦发银行等股份制银行落户我市。新设1到2家村镇银行，支持农村信用社组建农村商业银行。认真落实房地产调控政策，增加中小户型、中低价位普通商品房供给，促进房地产市场健康发展。着力发展体育健身、教育培训、家政服务、养老服务等社区服务业。大力发展科技服务、信息咨询、商务中介等新兴服务业。积极发展农村服务业。办好“万村千乡”市场工程，推进专业市场、农贸市场建设。继续做好家电、摩托车下乡和家电以旧换新工作。大力发展新农村现代流通服务网络，完善产销、科技、信息等社会化服务体系。

(四)着力发展现代农业，推进新农村建设。继续实施新农村建设提速工程，全面落实“两个规划”，促进城乡统筹发展。认真落实强农惠农政策。财政支出重点向农业农村倾斜，确保用于农业农村的总量、增量均有提高。稳步提高重要粮食品种最低收购价。继续扩大农业补贴规模，提高补贴标准。加大政策性金融对“三农”的支持力度。着力提高粮食生产能力。全面落实“粮食生产五年增产计划”，加快叶县、郏县和汝州市粮食核心区建设，粮食种植面积稳定在650万亩左右，总产保持在190万吨以上。加快调整农业结构。大力发展优质粮油、烟叶、林果等高效经济作物，抓好新一轮“菜篮子”工程，促进种植业结构优化。加快发展现代畜牧业，新建标准化养殖园区300个，积极引进或联建一批大型农畜产品加工企业。做大做强龙头企业，新增市级龙头企业10家，达到省级龙头企业标准3家以上，新增农民专业合作社50个。切实改善农村生产生活条件。大兴农田水利建设，新增有效灌溉面积10万亩，改造中低产田6万亩，治理水土流失面积60平方公里。落实农机购置补贴政策，玉米机收率达到80%以上。加强农村基础设施建设，新解决21万人的饮水安全问题，新建改建农村公路300公里，新建大中型沼气工程15座，新增沼气用户1万户，推进新一轮农村电网升级改造。加大扶贫开发力度，支持革命老区建设，完成5万贫困人口脱贫。加强农民工职业技能培训，实现转移就业6万人以上。在积极推进112个中心村建设的同时，再启动100个中心村的新村建设，继续实行领导联系分包责任制，深入开展“百企帮百村”活动，市财政安排1亿元奖励补助资金，加快建设进度。扎实做好南水北调干线征迁和移民安置工作。进一步提升县域经济发展水平。充分发挥县域经济统筹工业化、城镇化和农业现代化的重要载体作用，认真落实促进县域经济发展的政策措施，创新激励机制，加大扶持力度，引导城市资金、项目、技术、人才等要素向县域流动。各县(市)要依托资源禀赋和产业优势，培育壮大特色产业集群，增强县域经济综合实力。

(五)着力持续创建活动，提升城镇现代化水平。继续实施城镇化发展提速工程，加快城乡一体化进程。健全城乡规划体系。尽快完成平顶山城市新区规划报批，编制城市近期建设规划，实现市区建设用地控制性详细规划全覆盖；六县(市)、石龙区完成城区控制性详细规划编制，进一步推进城市扩容提质。强化规划执法，规范城乡建设行为。加大城市基础设施建设力度。按照新城区建设全面提速、老城区改造提档升级、新老城区同步发展的要求，加快中心城区建设步伐。老城区开工建设平安大道西延、光明路北延、一矿路改造、湛南路改造等21个项目；加快推进开源路高速引线改造、稻香路等8个续建项目；确保西环路、南环路、北环路、东环路改造项目建成通车；在人员密集、出行不便的地方，规划建设6座过街天桥；认真做好东环路南延、湛河治理等16个项目的前期工作；结合孟平铁路复线建设对市区23处铁路立交桥及引线进行升级改造。规划建设大南环、大北环，缓解中心城区交通压力，提高通行能力。全面推进城中村和旧城开发改造，新开工建设350万平方米，主体完工400万平方米；加快推进平安大道中段北部3.5平方公里旧城改造项目。加大城市管网改造力度，新建改造供水管道118公里、热力管道20公里，完成天然气置换人工煤气4万户。大力发展公共交通，更新公交车辆80台。新城区建设重点推进未来路、吉祥路、复兴路等工程，新增道路8.5公里。加快完善医院、商业网点、公交枢纽站等配套服务设施，不断提高综合承载能力。拓宽城市建设投融资渠道，采取发行城市建设债券、引导社会资金参与等办法，多渠道筹措建设资金。持续创建活动。继续加大创建力度，确保2012年创建为国家森林城市，2013年创建为国家卫生城市。深入开展城市精细化管理，启动数字城管建设。狠抓市容市貌和环境卫生综合整治，实行网格管理，划分责任区域，切实解决脏、乱、差、堵等问题。深入开展创建卫生达标庭院、园林小区活动。加大环城防护林、城郊森林体系和中心城市公共绿地建设力度，进一步提高城区森林覆盖率。争创和谐社区。全面落实和谐社区创建工作任务，加快推进行政服务、物业管理和文明教育进社区，逐步建立和完善新型社区服务体系，进一步提高服务水平。加快县城和小城镇建设。推进舞钢市城乡一体化进程。按照30万到40万人口规模对汝州市和郏县县城作扩张规划，对36个中心镇按每个不低于3万人的规模进行同步扩容。以县城扩容和中心镇建设为重点，加强基础设施建设，进一步增强县城和小城镇的吸纳承载能力。

(六)着力推进节能减排，建设生态文明。把节能减排和环境保护放在突出位置，统筹协调，综合施治，务求实效。强化资源集约节约利用。继续加大对高耗能、高耗材、高耗水行业的管控力度，做好重点领域、重点行业和重点企业的节能改造。持续推进公共机构节能工作。实施“关小上大”措施，加快淘汰落后产能。继续落实财税优惠政策和价格政策，开发利用节水、节电等节约新技术。积极推进建筑节能，开展既有住房供热计量及节能改造。落实最严格的耕地保护制度和最严格的节约用地制度，严禁违法用地，确保城乡发展特别是重点项目用地需求。大力发展循环经济，加快循环经济试点市建设。加强资源回收和综合利用，全市工业固体废弃物综合利用率达到76.5%以上。加强环境保护。深化大气环境综合整治，积极推进排污权交易，推广使用天然气等清洁燃料，城区大气环境质量优良天数保持在300天以上。加强白龟湖库区及上游环境综合整治，城市集中饮用水源地水质达标率100%。加大境内河流污染防治力度，全市出境水质达标率达到80%以上。加快林业生态建设。实施十大林业体系建设工程，新增造林24.9万亩，全市森林保有量达到366万亩；再创建1个省级林业生态县。通过持续努力，为全市人民创造一个天蓝、地绿、水清、宜于人居的生活环境。

(七)着力深化改革开放，增强经济发展活力。把改革开放作为推进科学发展的持久动力，形成有利于加快转变经济发展方式的体制机制。继续推进各项改革。加快推进体制创新，完成星峰集团、物资集团化轻公司、华靖物资中心等企业的改制。加大企业上市培育力度，加快易成新材料、圣光医药、平煤机等公司上市步伐。积极推进农村土地承包经营权流转，新增流转面积10万亩以上。加快集体林权制度改革，全面完成林改任务。大力推进教育、文化、医药卫生等领域的改革。积极推动企业战略合作。研究制定扶持政策，着力推动六大支柱产业同大型央企、世界500强企业的战略合作。巩固扩大平高集团与国家电网公司战略重组成果，加快输配电装备制造产业化。推动平煤机与国际煤机巨头美国久益公司合作，打造高端采矿设备研发制造基地。着力提高招商选资实效。采取领导带队驻地招商、商会招商、委托招商等多种形式，持续开展大招商活动，引导支持各类产业集聚区发挥自身优势，承接产业转移，力争引进省外资金和市外资金均增长20%以上。同时，继续落实鼓励出口的各项政策，加大对重点出口企业和出口产品的扶持力度，努力扩大外贸出口。下大力气优化发展环境。坚持诚实守信、有诺必践，确保开放招商项目零障碍落户，创造良好的投资环境。全面清理规范各种收费项目，规范对企业的各种检查行为，健全投诉受理机制，严厉查处“四乱”行为，为企业营造良好的经营环境。大力发展非公有制经济。认真落实促进非公有制经济发展的政策措施，通过政策、资金的引导，留住并激活民间资本在我市创业发展，促进中小企业做大做强。稳妥推进设立小额贷款公司，办好市中小企业金融超市，努力缓解贷款难问题。

(八)着力保障和改善民生，促进社会和谐。进一步加大投入，继续实施“十大民生工程”，让改革发展的成果更多地惠及人民群众。千方百计扩大就业。实施更加积极的就业政策，提高中小企业、劳动密集型企业和服务业吸纳就业的能力。扶持自主创业和自谋职业。突出抓好高校毕业生、农村转移劳动力、城镇就业困难人员、退役军人的就业问题，确保城镇新增就业7万人以上，其中失业人员再就业2.5万人，动态消除“零就业”家庭。努力提高社会保障水平。进一步完善企业工资集体协商和工资支付保障制度，提高企业退休人员基本养老金，将未参保集体企业退休人员纳入基本养老保险。深化城乡低保规范化建设，提高低保标准和补助水平，提高农村五保对象供养标准和集中供养率，切实保障困难群众基本生活。逐步扩大社会保险覆盖范围，再争取1个县纳入国家新农保试点。完善城乡医疗救助和临时救助制度，提高救助标准，扩大救助覆盖面。大力加强保障性住房建设。优先保证用地供应，加强市、县(市、区)财政支付保障，新开工建设廉租房42万平方米、公共租赁房14.7万平方米、经济适用房57.2万平方米，提供保障性住房1.8万套，发放住房租赁补贴2000万元，为2300户农村贫困家庭实施危房改造。全面发展社会事业。认真落实中长期教育发展规划。大力推进义务教育均衡发展，加快新一轮中小学布局结构调整，启动市四十一中改扩建项目，确保3所在建小学秋季投入使用、6所新建小学顺利开工，3年内解决大班额问题。制定实施学前教育三年行动计划，市内三区新建8所幼儿园，其他县(市)新建19所乡(镇)中心幼儿园。规划建设市教育园区。强力实施职教攻坚计划，加快城市职业教育改革实验区建设，建成专业实训基地6个。继续实施普通高中家庭困难学生资助制度，扩大中等职业教育免学费范围。深入实施中小学校舍安全工程，完成5.4万平方米的校舍加固或重建。支持高等教育走特色发展、内涵发展之路，提高教育教学质量。贯彻落实人才发展规划，壮大各类人才队伍。繁荣文化事业，启动市图书馆改造、广播电视安全播出监测中心等项目，实施好乡(镇)综合文化站、文化信息资源共享、农家书屋等项目，高标准完成广播电视“村村通”、农村(校园)公益电影放映、舞台艺术送农民等工程。加大对重点文物保护单位的抢救修缮力度。规划建设老干部活动中心和老干部大学。加强医疗卫生基础设施建设，扎实推进区域卫生信息平台、市精神卫生中心、市职业病防治综合楼等项目，继续抓好传染病防治，不断提高群众健康水平。积极发展体育事业，举办市八运会，做好新城区体育馆建设规划。在全省率先推进残疾人社会保障体系和服务体系建设，确保创建为全国残疾人工作示范市。积极发展社会福利和慈善事业。统筹做好人口计生工作，完善利益导向机制，稳定低生育水平，确保在全国、全省的先进位次。进一步强化安全监管。认真落实安全生产行政首长负责制，进一步完善安全生产责任体系。突出抓好以煤矿为重点的安全生产管理，确保兼并重组煤矿停得住、管得好。持续深化非煤矿山、道路交通、消防、食品药品、危险化学品、烟花爆竹、建筑施工、民爆器材、特种设备等重点行业、重点领域的专项整治，严厉打击非法违法生产经营建设行为，坚决遏制重特大事故发生。加强和改进社会管理。深入推进社会矛盾化解、社会管理创新、公正廉洁执法三项重点工作，健全信访工作责任制，畅通群众利益诉求渠道，认真解决群众反映的问题。大力推进“六五”普法和依法治市，切实加强人民调解、法律援助、法律服务工作。完善社会治安防控体系，严密防范、严厉打击各类违法犯罪活动，不断增强人民群众的安全感。支持工会、共青团、妇联等人民团体充分发挥桥梁纽带和参与社会事务管理的作用。完善村民自治，加强村务公开、民主管理。深入开展“扫黄打非”，净化社会环境。积极拓展群众性精神文明创建活动，加强青少年思想道德建设，不断提高城乡文明程度。

加强国防教育和动员，支持驻平解放军、武警部队、民兵预备役部队和人民防空建设；深入开展双拥共建活动，巩固和发展军政军民团结，确保创建全国双拥模范城“四连冠”。加强统计工作，搞好第六次人口普查。继续做好民族、宗教、外事、侨务、对台、史志、气象、通信、烟草、档案、盐业、新闻出版、国家安全、防震减灾等工作。

(九)着力加强政府自身建设，提高推动科学发展的能力。实现发展目标任务，政府肩负着重要责任。我们一定勤勉尽责，永不懈怠，努力建设人民满意的政府。

坚持执政为民。时刻牢记为人民服务的宗旨，用人民赋予的权力为人民谋福祉。作决策、编规划、办事情始终站在群众角度换位思考，一心想着群众，真心为了群众。充分发挥政府门户网站、市长热线、政风行风热线联系群众的桥梁纽带作用，认真受理和解决群众反映的问题。办好各级行政审批服务中心，方便企业和群众办事。建立健全应急管理机制，增强处置突发事件的能力。加强对政府公务人员的教育和管理，继续开展争做“人民满意的公务员”活动。

严格依法行政。认真执行人大及其常委会的决议、决定，自觉接受人大及其常委会的法律监督、工作监督和政协的民主监督，广泛听取民主党派、工商联、无党派人士、人民团体的意见和建议，重视新闻监督和网络监督。认真办理人大代表建议和政协提案，不断提高落实率和满意度。严格按照法定权限和程序行使权力、履行职责，健全重大事项公众参与、专家论证、风险评估、合法性审查、集体讨论决定等制度。强化行政执法责任制，做到有权必有责、用权受监督。扩大政府信息公开范围，让权力在阳光下运行。

注重实干实效。坚持以领导方式转变加快发展方式转变，学先进、比创新、看实效，切实提升各级干部的工作推动力和执行力，不找借口、不打折扣，说到做到、说好做好。进一步提高行政效能，改进会风文风，严格控制各类检查评比，减少不必要的应酬和事务性活动，把更多的心思和精力放在调查研究、谋划发展、推动工作上，确保各项目标任务落到实处、见到实效，真正做出经得起实践、历史、人民检验的实绩。

做到廉洁从政。严格落实党风廉政建设责任制，加快推进惩治和预防腐败体系建设，完善工程建设、土地管理、政府采购等制度，加强监察、审计等专项监督，做到关口前移。继续保持惩治腐败的高压态势，严肃查处各类违法违纪案件。坚持勤俭行政，反对铺张浪费。领导干部带头执行廉洁自律的各项规定，要求别人做到的，自己首先做到，要求别人不做的，自己坚决不做，塑造风清气正的政务环境。

各位代表!

“十二五”的蓝图已经绘就，新一轮发展的号角已经吹响。让我们在省委、省政府和市委的正确领导下，深入贯彻落实科学发展观，按照“学先进、比创新、看实效”的要求，满怀激情，顽强拼搏，同心协力，扎实工作，以优异的成绩向建党90周年献礼，为“率先实现崛起、提前全面小康”而努力奋斗!
